# Temporal trends in female breast cancer mortality in Brazil and correlations with social inequalities: ecological time-series study

**DOI:** 10.1186/s12889-015-1445-7

**Published:** 2015-02-07

**Authors:** Carolina Maciel Reis Gonzaga, Ruffo Freitas-Junior, Maria-Paula Curado, Ana-Luiza Lima Sousa, José-Augusto Souza-Neto, Marta Rovery Souza

**Affiliations:** Health Sciences, Federal University of Goiás (UFG), Goiânia, Brazil; Department of Obstetrics and Gynecology, Federal University of Goiás (UFG), Goiânia, Brazil; Hospital Araújo Jorge, Goiás Anticancer Association (ACCG), Goiânia, Brazil; International Prevention Research Institute (IPRI), Lyon, France; Federal University of Goiás (UFG), Goiânia, Brazil; Institute of Tropical Pathology and Public Health, Federal University of Goiás (UFG), Goiânia, Brazil; Alameda das Rosas, 533, Setor Oeste, 74110-060 Goiânia, GO Brazil

**Keywords:** Breast cancer, Mortality, Trends, Brazil, Socioeconomic factors

## Abstract

**Background:**

Breast cancer is the most common cause of death from cancer in women in less developed regions. Therefore, the objective of this study was to provide data on the temporal trends in female breast cancer mortality between 1990 and 2011 and to evaluate its association with the social inequalities present in Brazil.

**Methods:**

Breast cancer mortality data and estimates for the resident population were obtained from the Brazilian National Health Service database for the 1990–2011 period. Age-standardized mortality rates were calculated (20–39, 40–49, 50–69 and ≥70 years) by direct standardization using the 1960 standard world population. Trends were modeled using joinpoint regression model and linear regression. The Social Exclusion Index and the Human Development Index were used to classify the 27 Brazilian states. Pearson’s correlation was used to describe the association between the Social Exclusion Index and the Human DeveIopment and the variations in mortality rates in each state.

**Results:**

Age-standardized mortality rates in Brazil were found to be stable (annual percent change [APC] = 0.3; 95% CI: −0.1 – 0.7) between 1994 and 2011. Considering the Brazilian states, significant decreases in mortality rates were found in Rio Grande do Sul, Rio de Janeiro and São Paulo. Increases in mortality rates were most notable in the states of Maranhão (APC = 11.2; 95 %CI: 5.8 – 16.9), Piauí (APC = 9.8; 95% CI: 7.6 – 12.1) and Paraíba (APC = 9.3; 95% CI: 6.0 – 12.8). There was a statistically significant correlation between Social Exclusion Index and a change in female breast cancer mortality rates in the Brazilian states between 1990 and 2011 and between Human Development Index and mortality between 2001 and 2011.

**Conclusions:**

Female breast cancer mortality rates are stable in Brazil. Reductions in these rates were found in the more developed states, possibly reflecting better healthcare.

## Background

Breast cancer is the most common cause of death from cancer in women in less developed regions (324,000 deaths; 14.3% of the total) and the second cause of death from cancer in women after lung cancer in the more developed regions (198,000 deaths). Despite the high incidence of the disease in the developed world, mortality rates are much lower because of favorable survival rates [1]. Data collected in the United States over the past two decades show that breast cancer mortality rates have decreased by more than 30% since their peak in 1991; nevertheless, breast cancer is still the most common cause of death from cancer in women of 20 to 59 years of age [2].

In Brazil, mortality rates range from 14 per 100,000 in the south and southeast to 6.6 per 100,000 in the north of the country. Nonetheless, a significant decrease has been seen in the southeast since 1997 (APC = −0.9%) and a non-significant decrease in the south of the country. In the other regions, although rates were lower, there was a tendency towards an increase. The highest mortality rates were found in the state of Rio de Janeiro (18.8 in 1994) and in the Federal District (18.4 in 2006) [3]. In urban areas, a significant decrease in mortality was found in five cities compared to only one rural district. Breast cancer mortality increased in the majority of rural regions in Brazil with the exception of some areas in the south and southeast of the country. A possible reason for this disparity may be that access to treatment is more difficult for patients living in rural areas [4].

Advances in treatment have considerably improved the prognosis of breast cancer in recent decades, with survival rates reaching approximately 90% for white women in the United States [2]. Paradoxically, improvements in treatment increased inequalities in health resources between women living in rich countries and those living in middle to low income countries. The most important factors are the availability of drugs at an accessible cost and of centers of excellence to guarantee the efficacy and safety of treatment [5].

International initiatives have highlighted the need to improve breast cancer treatment in developing countries [6], with the objective of decreasing mortality by reducing disparities in the access to treatment and diagnostic methods. The high mortality rates in these countries are known to be a consequence of the fact that diagnosis is made at an advanced stage and that resources for treatment are scarce.

The Brazilian population varies insofar as their ethnic origin, culture and socioeconomic conditions are concerned. For this reason, providing high quality healthcare throughout the entire country is a challenge [7], and there is a need for further epidemiological studies on breast cancer. Therefore, the objective of this study was to provide data on the temporal trends in female breast cancer mortality between 1990 and 2011 and to evaluate its association with the social inequalities present in Brazil.

## Methods

Cancer mortality data and estimates for the resident population from 20 to 39, 40 to 49, 50 to 69 and ≥70 years of age for the 1990 to 2011 period were obtained from the Ministry of Health’s database (DATASUS) [8]. Breast cancer deaths were coded according to the International Classification of Diseases (ICD). The ninth revision of the ICD (ICD-9) was used for the 1990–1995 period (ICD code 174), while the 10^th^ revision was used for the 1996–2011 period (ICD code C50). Age-standardized mortality rates for women of 20 years of age or more were obtained by direct standardization based on the 1960 world standard population. Variations in mortality were modeled using standard linear regression methods, with the age-standardized mortality coefficient as the dependent variable and the calendar year as the independent variable.

Trends in age-adjusted breast cancer death rates were estimated using joinpoint regression, which involves fitting a series of joined straight lines on a logarithmic scale to the trends in the annual age-adjusted mortality rates [9] using the Joinpoint Regression Program (version 4.0). A maximum of 3 joinpoints was allowed in models for the period 1990 through 2011. The resulting trends were described by the slope of the line segment or the annual percent change (APC). The average APC (AAPC) was estimates as a weighted geometric average of the APCs, with the weights equal to the length of each line segment during the prespecified, fixed interval. The 95% confidence intervals (95% CIs) were calculated for each estimated APC. If these intervals excluded zero, then the APCs were statistically significant (p < .05).

**Figure 1:**
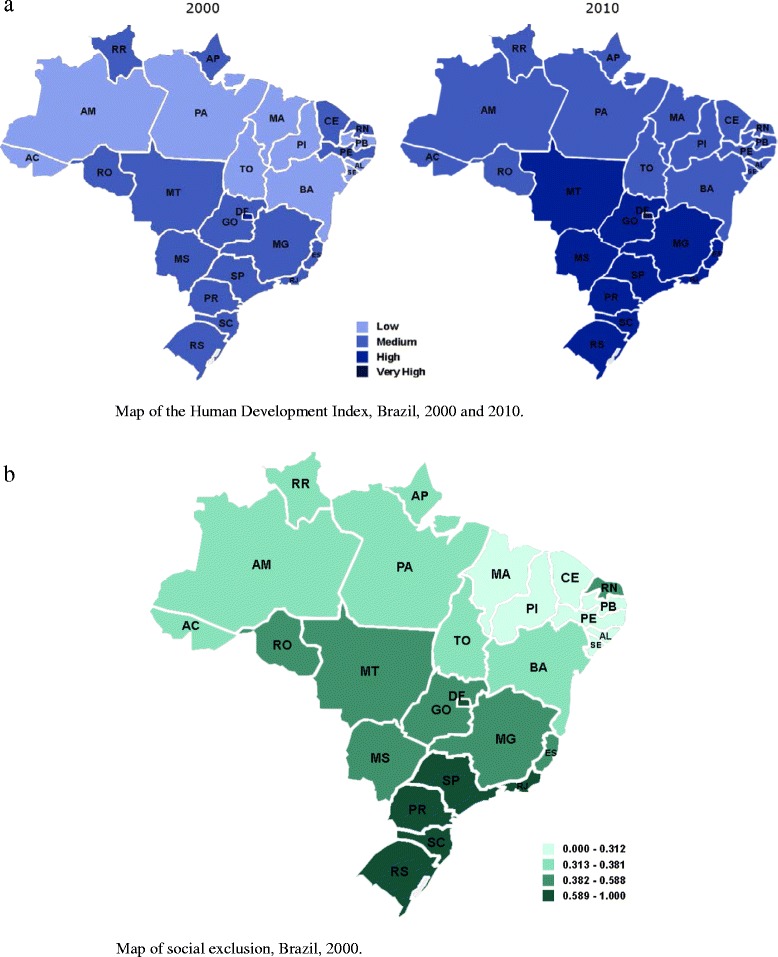
**Map of Brazil. a**. Human Development Index, 2000 and 2010. **b**. Social Exclusion Index, 2000. The lowest SEI was in the state of Maranhão (0.197) and the highest in the Federal District (0.850) *Acre (AC), Alagoas (AL), Amapá (AP), Amazonas (AM), Bahia (BA), Ceará (CE), Distrito Federal (DF), Espírito Santo (ES), Goiás (GO), Maranhão (MA), Mato Grosso (MT), Mato Grosso do Sul (MS), Minas Gerais (MG), Pará (PA), Paraíba (PB), Paraná (PR); Pernambuco (PE), Piauí (PI), Rio de Janeiro (RJ), Rio Grande do Norte (RN), Rio Grande do Sul (RS), Rondônia (RO), Roraima (RR), Santa Catarina (SC), São Paulo (SP), Sergipe (SE), Tocantins (TO)*.

The Social Exclusion Index (SEI) and the Human Development Index (HDI), which ranges from 0 (worst) to 1 (best), were used to classify the 27 Brazilian states. The human development index (HDI) is a composite statistic of life expectancy, education and income indices used to rank countries into four tiers of human development, taking into consideration a concept of well-being viewed in terms of capability. The lowest HDI was in the state of Alagoas (0.631), while the highest was in the Federal District (0.824) (Figure 1a). The SEI was based on three dimensions: suitable life conditions, knowledge and youth vulnerability, as described previously [10]. The lowest SEI was in the state of Maranhão (0.197) and the highest in the Federal District (0.850) (Figure 1b).

Pearson correlation coefficients were used to describe the correlation between the SEI and the variations in mortality rates in each Brazilian state for the entire evaluation period, the HDI (2000) for the 1990–2000 period and the HDI (2010) for the 2001–2010 period. For all the statistical tests, a type 1 (α) error of 5% was adopted.

This project has been approved by the Ethics Committee on Human and Animal Research of the Hospital of the Federal University of Goiás (Protocol number 75793).

## Results

**Figure 2:**
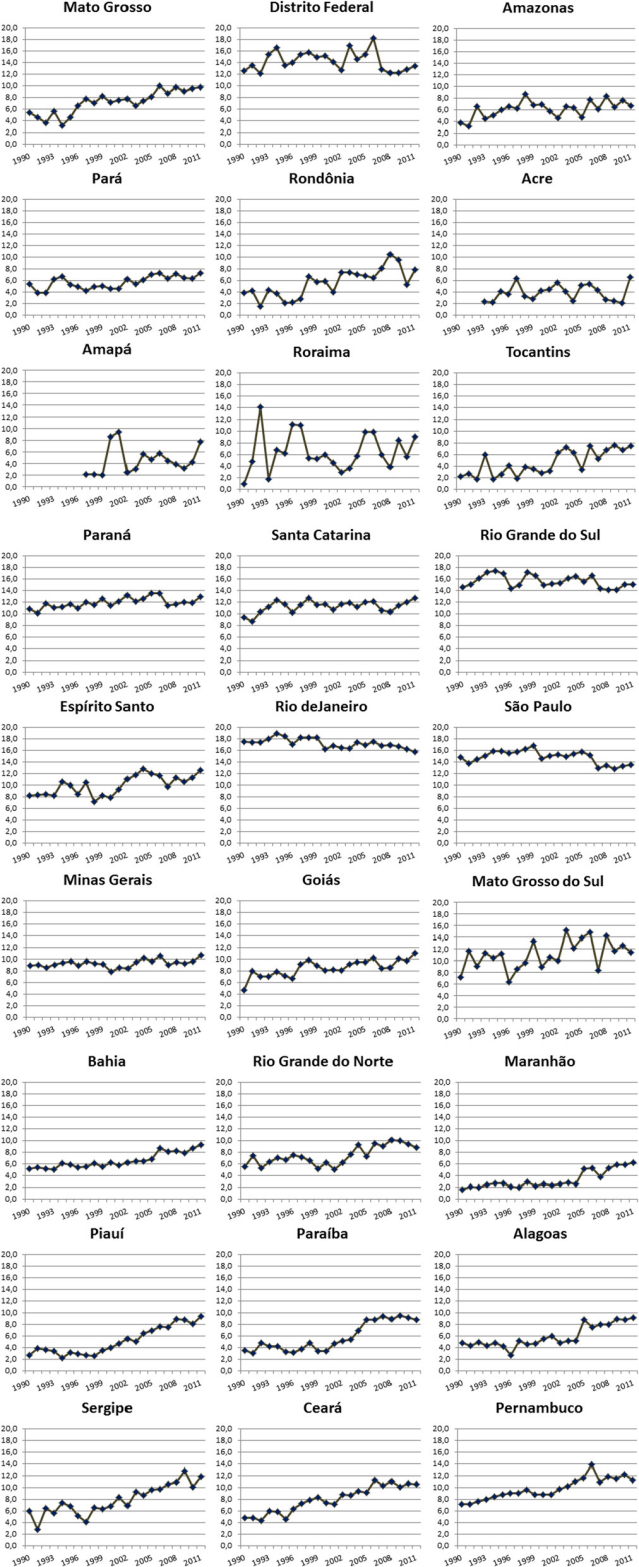
**Female breast cancer mortality rates in the states of Brazil from 1990 to 2011.**

Between 1990 and 2011, 195,596 deaths from breast cancer were registered for Brazilian women of 20 years of age or more. By age group, the highest rates were in the group of Brazilian women of 70 years of age or older (Table 1). In Brazil, the highest age-adjusted mortality rate was in 2006 (12.7/100,000) and the lowest in 1991 (10.2/100,000). The highest mortality rate in the entire period evaluated was found in the state of Rio de Janeiro (17.2/100,000) and the lowest in the state of Maranhão (3.2/100,000) (Figure 2).

**Table 1:** **Age-specific and age-standardized world female breast cancer mortality rates according to year: Brazil, 1990-2011**

	**Mortality rate (per 100.000)**				
**Year**	**Age-specific**				**Age-adjusted**
	**20-39 years**	**40-49 years**	**50-69 years**	**≥70 years**	**≥20 years**
1990	2.5	16.0	34.0	56.7	10.3
1991	2.3	16.3	33.5	56.7	10.2
1992	2.4	16.5	34.6	57.1	10.5
1993	2.4	17.8	35.9	62.3	11.0
1994	2.7	18.5	37.5	65.5	11.6
1995	2.7	17.7	36.9	65.6	11.4
1996	2.5	16.3	34.5	62.0	10.7
1997	2.4	17.9	36.5	65.5	11.3
1998	2.6	17.7	37.4	71.7	11.7
1999	2.7	17.7	37.0	72.8	11.7
2000	2.5	16.7	33.8	62.5	10.6
2001	2.5	16.7	35.3	64.3	10.9
2002	2.6	16.7	37.0	64.8	11.3
2003	2.5	17.5	36.9	70.7	11.5
2004	2.6	17.7	38.9	72.2	11.9
2005	2.5	18.3	39.2	74.5	12.1
2006	2.6	18.1	41.4	79.8	12.7
2007	2.6	16.5	35.4	68.2	11.1
2008	2.8	17.1	37.2	70.1	11.6
2009	2.9	17.0	36.0	69.1	11.4
2010	2.9	17.2	37.2	69.4	11.6
2011	3.0	17.9	38.0	72.2	12.0

A statistically significant increase in breast cancer mortality rates in Brazil, was found for the following age groups: 20–39 years (APC = 0.7; 95% CI: 0.3 – 1.1, p < 0.01), 50–69 years (APC = 0.4; 95% CI: 0.1 – 0.8, p < 0.01) and 70 years of age or older (APC = 1.0; 95% CI: 0.6 – 1.5, p < 0.01). There was also a trend towards an increase in the 40–49 years age group; however this increase was not statistically significant: (APC = 0.2; 95% CI: −0.0 – 0.5, p = 0.1).

Female breast cancer mortality rates have remained stable (APC = 0.3; 95% CI: −0.1 – 0.7) in Brazil, between 1994 and 2011 (Table 2). A statistically significant decrease in mortality was found in the states of Rio Grande do Sul, Rio de Janeiro and São Paulo (Table 2).

**Table 2:** **Temporal trends in mortality from female breast cancer in the states of Brazil between 1990 and 2011**

**States**	**Trend 1**			**Trend 2**			**AAPC**
	**Years**	**APC**	**(95% CI; p-value)**	**Years**	**APC**	**(95% CI; p-value)**	**2002-2011**
Paraná	1990-2005	1.2*	(0.6 to 1.9; p < 0.01)	2005-2011	−1.2	(−3.8 to 1.4; p = 0.33)	−0.4
Santa Catarina	1990-1994	6.6*	(0.2 to 13.5; p = 0.04)	1994-2011	0.0	(−0.7 to 0.7; p = 0.92)	0.0
Rio Grande do Sul	1990-1993	4.6	(−4.5 to 14.6; p = 0.31)	1993-2011	−0.7*	(−1.3 to −0.1; p = 0.01)	−0.7*
Espírito Santo	1990-2011	1.8*	(0.9 to 2.8; p < 0.01)				1.8*
Rio de Janeiro	1990-2011	−0.5*	(−0.8 to −0.2; p < 0.01)				−0.5*
São Paulo	1990-1998	1.7*	(0.0 to 3.4; p = 0.04)	1998-2011	−1.7*	(−2.4 to −0.9; p < 0.01)	−1.7*
Minas Gerais	1990-2011	0.5*	(0.0 to 1.0; p = 0.04)				0.5*
Goiás	1990-2011	2.2*	(1.3 to 3.1; p < 0.01)				2.2*
Mato Grosso do Sul	1990-2011	1.7*	(0.2 to 3.2; p = 0.02)				1.7*
Mato Grosso	1990-2011	4.2*	(2.9 to 4.5; p < 0.01)				4.2*
Distrito Federal	1990-2011	−0.2	(−1.0 to 0.7; p = 0.68)				−0.2
Amazonas	1990-2011	2.1*	(0.6 to 3.7, p < 0.01)				2.1*
Pará	1990-2011	2.2*	(1.0 to 3.3, p < 0.01)				2.2*
Rondônia	1990-2011	6.0*	(3.4 to 8.7, p < 0.01)				6.0*
Acre	1993-2011	0.8	(−2.6 to 4.2; p = 0.64)				0.8
Amapá	1997-2011	4.6	(−1.9 to 11.6, p = 0.15)				4.6
Roraima	1990-2011	2.8	(−1.6 to 7.4; p = 0.20)				2.8
Tocantins	1990-2011	6.3*	(3.8 to 8.9; p < 0.01)				6.3*
Bahia	1990-2001	1.2	(−0.1 to 2.6; p = 0.06)	2001-2011	4.4*	(2.9 to 6.0; p < 0.01)	4.4*
Rio Grande do Norte	1990-2011	2.4*	(1.3 to 3.6; p < 0.01)				2.4*
Maranhão	1990-2002	2.6	(−0.7 to 5.9; p < 0.11)	2002-2011	11.2*	(5.8 to16.9; p < 0.01)	11.2*
Piauí	1990-1997	−1.6	(−7.3 to 4.5; p = 0.58)	1997-2011	9.8*	(7.6 to 12.1; p < 0.01)	9.8*
Paraíba	1990-1999	0.8	(−3.9 to 5.8; p = 0.72)	1999-2011	9.3*	(6.0 to 12.8; p < 0.01)	9.3*
Alagoas	1990-2011	4.1*	(2.7 to 5.5; p < 0.01)				4.1*
Sergipe	1990-2011	4.8*	(3.3 to 6.4; p < 0.01)				4.8*
Ceará	1990-2011	4.5*	(3.7 to 5.3; p < 0.01)				4.5*
Pernambuco	1990-2011	2.6*	(2.0 to 3.1; p < 0.01)				2.6*
Brazil	1990-1994	2.5	(−1.3 to 6.5; p = 0.18)	1994-2011	0.3	(−0.1 to 0.7; p = 0.13)	0.3

*Abbreviations: AAPC* average annual percent change; *APC* annual percent change.

Joinpoint analyses with up to 1 joinpoints yielding up to 2 trend segments (Trends 1–2).

The AAPC is a weighted average of the APCs that is calculated by joinpoint regression.

Standardized mortality rate per 100,000 women.

*Statistically significant; p = 0.05.

Increasing trends in mortality rates were most notable in the states of Maranhão (APC = 11.2; 95% CI: 5.8 – 16.9) between 2002 and 2011, Piauí (APC = 9.8; 95% CI: 7.6 – 12.1), between 1997 and 2011 and Paraíba (APC = 9.3; 95% CI: 6.0 – 12.8) between 1999 and 2011 (Table 2).

**Figure 3:**
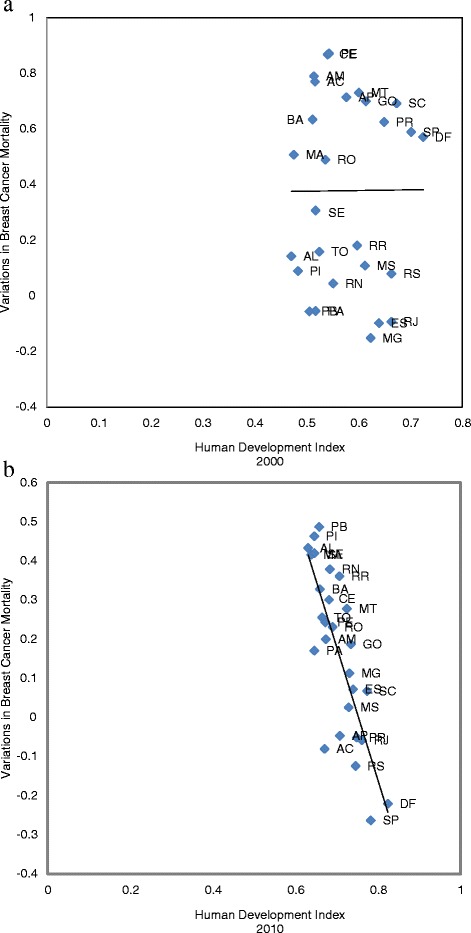
**Correlation between the variations in breast cancer mortality rates for women in Brazil. a**. Human Development Index (2000) from 1990 to 2000. (r = 0.28; p = .156). **b**. Human Development Index (2010) from 2001 to 2011. (r = -0.79; p<.001). *Acre (AC), Alagoas (AL), Amapá (AP), Amazonas (AM), Bahia (BA), Ceará (CE), Distrito Federal (DF), Espírito Santo (ES), Goiás (GO), Maranhão (MA), Mato Grosso (MT), Mato Grosso do Sul (MS), Minas Gerais (MG), Pará (PA), Paraíba (PB), Paraná (PR); Pernambuco (PE), Piauí (PI), Rio de Janeiro (RJ), Rio Grande do Norte (RN), Rio Grande do Sul (RS), Rondônia (RO), Roraima (RR), Santa Catarina (SC), São Paulo (SP), Sergipe (SE), Tocantins (TO)*.

**Figure 4:**
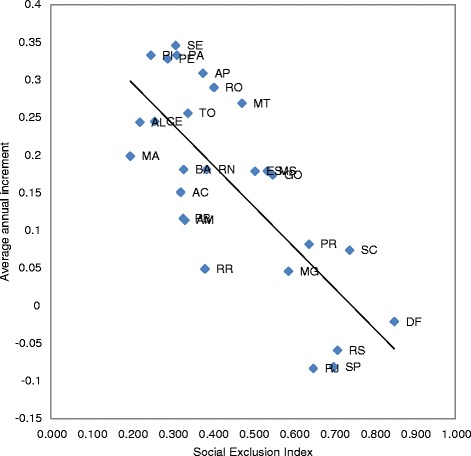
**Correlation between the Social Exclusion Index and the variations in breast cancer mortality rates for women in Brazil from 1990 to 2011.** (r = -0.75; p<0.001). Acre (AC), Alagoas (AL), Amapá (AP), Amazonas (AM), Bahia (BA), Ceará (CE), Distrito Federal (DF), Espírito Santo (ES), Goiás (GO), Maranhão (MA), Mato Grosso (MT), Mato Grosso do Sul (MS), Minas Gerais (MG), Pará (PA), Paraíba (PB), Paraná (PR); Pernambuco (PE), Piauí (PI), Rio de Janeiro (RJ), Rio Grande do Norte (RN), Rio Grande do Sul (RS), Rondônia (RO), Roraima (RR), Santa Catarina (SC), São Paulo (SP), Sergipe (SE), Tocantins (TO).

Linear regression models showed disparities in female breast cancer mortality trends in Brazil. There was no statistically significant correlation between HDI and a change in the female breast cancer mortality rates in Brazil (r = 0.28; p = .156) between 1990 and 2000 (Figure 3a). However, for the 2001–2011 period, a statistically significant correlation was found between HDI and the change in female breast cancer mortality rates in the country (r = −0.79; p < .001) (Figure 3b) and also between SEI and mortality (r = −0.75; p < 0.001) for the overall period (Figure 4).

## Discussion

To the best of our knowledge, this is the first report to analyze the temporal trends in female breast cancer mortality in Brazil and to correlate them with social inequalities. The study of mortality trends is useful for monitoring changes in the epidemiological profile of the population.

The female breast cancer mortality rates in Brazil described in the present study are similar to rates found earlier in Chile, Costa Rica, Cuba, Puerto Rico and Venezuela [11], values expected for developing countries [1]. However, they are higher than rates found in Colombia, Ecuador and Mexico (<10/100,000) [11]. Female breast cancer mortality trends have remained stable in the country since 1994, this finding being in agreement with reports from another study (APC = 0.4%) [3].

A marked disparity was found between the different Brazilian states with respect to mortality rates, which ranged from 2-5/100,000 in the less developed areas of the country to 12-18/100,000 in the more developed regions. Although rates were higher in these states, there was a significant decrease in mortality rates in Rio Grande do Sul, Rio de Janeiro and São Paulo, similar to that seen in developed countries such as the US (APC = −1.9%) [2] and Portugal (APC = −0.9%) [12], and also in some developing countries such as Singapore (APC = −1.50%) [13]. Nevertheless, the mortality rates here are lower than those found in these countries.

One possible hypothesis for the lower breast cancer mortality rates found in Brazil may be under-notification. It is known that the cancer registries cover only 6% of the Latin American population in comparison to 96% in the US and 32% in Europe [14]. There are 17 population-based cancer registries in the country, 16 of which are located in state capital cities. Data collection varies from registry to registry and also from one year to another within a single registry [8].

Another hypothesis for the patterns of inequality in health found in Brazil with respect to breast cancer mortality may be the lack of available resources for treatment in the less developed states or the inaccessibility of the majority of the population to treatment. In some cases, the situation would be comparable to that found in Nigeria where there is no specific screening program within the national healthcare system and only two hospitals offering tertiary treatment (radiotherapy and chemotherapy) [15]. Other problems identified in Brazil include: a lack of information on the disease, the time interval between the first signs/symptoms and first consultation (ranging from 1 to 60 months), delays in diagnosing and treating breast cancer and the time between histopathological diagnosis and the beginning of treatment.

The Amazona Project conducted by the Brazilian Group for Studies in Breast Cancer (GBECAM) collected data from 28 centers, including 4,912 cases of patients diagnosed with breast cancer in 2001 and 2006, representing all the geographical regions of the country and all socioeconomic levels. That study showed the disease to be more advanced at diagnosis in the women treated in public institutions, who had less access to modern therapies and poorer survival compared to patients treated in private institutions [16].

This study evaluated data from nationwide Brazilian surveys, databases and local studies, and its results highlight the gaps in information and the need to acquire further knowledge and to conduct more studies on various aspects. This report provides further information on female breast cancer mortality in Brazil, thus permitting actions to be implemented by enabling predictions to be made of what is required by women and by healthcare services. Although the treatment options for breast cancer are effective, they unfortunately remain inaccessible to many women living in developing areas. Lack of care has resulted in unnecessary deaths. The unequal distribution of resources for breast cancer care and control for women living in the same country is unacceptable.

## Conclusions

Although there has been a trend toward stabilization in female breast cancer mortality rates in Brazil, when the mortality rate of each state is analyzed individually, considerable inequalities are found, with rate decline or stabilization in states with higher socioeconomic levels and a substantial rate increase in those with lower socioeconomic levels. Reductions in these rates were found in the more developed states, possibly reflecting better healthcare.

## Limitations

The use of the mortality data from the mortality data system is subject to correction due to an under-registration of deaths that is unfortunately common in less developed areas. The demographic database may contain errors that are inherent to data collection or to the methodology used to calculate population-based estimates. With respect to the breast cancer mortality rate, there are always limitations to its use when a considerable proportion of deaths take place without medical care or because of other poorly defined causes.

## Abbreviations

APC: Annual percent change
AAPC: Average annual percent change
DATASUS: Ministry of Health’s database
ICD: International Classification of Diseases
CI: Confidence intervals
SEI: The Social Exclusion Index
HDI: Human Development Index
GBECAM: Brazilian Group for Studies in Breast Cancer
